# Next-generation sequencing diagnostics of bacteremia in septic patients

**DOI:** 10.1186/s13073-016-0326-8

**Published:** 2016-07-01

**Authors:** Silke Grumaz, Philip Stevens, Christian Grumaz, Sebastian O. Decker, Markus A. Weigand, Stefan Hofer, Thorsten Brenner, Arndt von Haeseler, Kai Sohn

**Affiliations:** Fraunhofer IGB, Nobelstr. 12, 70569 Stuttgart, Germany; IGVP, University of Stuttgart, Nobelstr. 12, 70569 Stuttgart, Germany; Department of Anesthesiology, Heidelberg University Hospital, Im Neuenheimer Feld 110, 69120 Heidelberg, Germany; Center for Integrative Bioinformatics Vienna, Max F. Perutz Laboratories, University of Vienna, Medical University of Vienna, Vienna, Austria; Bioinformatics and Computational Biology, Faculty of Computer Science, University of Vienna, Vienna, Austria

**Keywords:** Sepsis, Diagnostics, Next-generation sequencing, Circulating nucleic acids

## Abstract

**Background:**

Bloodstream infections remain one of the major challenges in intensive care units, leading to sepsis or even septic shock in many cases. Due to the lack of timely diagnostic approaches with sufficient sensitivity, mortality rates of sepsis are still unacceptably high. However a prompt diagnosis of the causative microorganism is critical to significantly improve outcome of bloodstream infections. Although various targeted molecular tests for blood samples are available, time-consuming blood culture-based approaches still represent the standard of care for the identification of bacteria.

**Methods:**

Here we describe the establishment of a complete diagnostic workflow for the identification of infectious microorganisms from seven septic patients based on unbiased sequence analyses of free circulating DNA from plasma by next-generation sequencing.

**Results:**

We found significant levels of DNA fragments derived from pathogenic bacteria in samples from septic patients. Quantitative evaluation of normalized read counts and introduction of a sepsis indicating quantifier (SIQ) score allowed for an unambiguous identification of Gram-positive as well as Gram-negative bacteria that exactly matched with blood cultures from corresponding patient samples. In addition, we also identified species from samples where blood cultures were negative. Reads of non-human origin also comprised fragments derived from antimicrobial resistance genes, showing that, in principle, prediction of specific types of resistance might be possible.

**Conclusions:**

The complete workflow from sample preparation to species identification report could be accomplished in roughly 30 h, thus making this approach a promising diagnostic platform for critically ill patients suffering from bloodstream infections.

**Electronic supplementary material:**

The online version of this article (doi:10.1186/s13073-016-0326-8) contains supplementary material, which is available to authorized users.

## Background

Sepsis remains a challenge in intensive care medicine, its incidence increasing continuously over the past decades [1, 2]. Despite massive efforts in sepsis research, new therapeutic approaches are rare and mortality in patients with septic shock still remains unacceptably high [1, 2]. In addition to an early focus on control, recent guidelines recommend the initiation of an empiric antibiotic therapy as early as possible (preferably within 1 h) following diagnosis of sepsis [3]. However, the identification of the causative pathogen as well as its resistance pattern is crucial for early optimization of the antimicrobial treatment regime. In this context, culture-based diagnostic procedures (e.g., blood cultures) represent the standard of care, although they are associated with relevant limitations [3, 4]: (i) depending on microbiological growth, it often takes up to 5 days for results to become available; and (ii) culture-based diagnostic procedures often reveal false negative results due to the administration of an empiric antibiotic therapy. Accordingly, patients suffering from septic disease are at high risk for antimicrobial overtreatment, antibiotics-related toxicity, and the selection of multi-drug resistant pathogens due to the inadequate and prolonged use of broad spectrum antibiotics. In this context, culture-independent molecular diagnostic procedures (e.g., PCR-based techniques) have already been introduced for the identification of the causative pathogen in infected patients [5–9]. However, the occurrence of ambiguous results as well as limitations in the quantitative measurement of the bacterial load in patients’ samples and detection of antibiotic resistance markers are known limitations of these PCR-based diagnostic approaches. Therefore, the concept of our work was to develop an alternative diagnostic platform for the identification of infectious microorganisms based on unbiased sequence analyses of circulating cell-free DNA (cfDNA) from plasma samples of septic patients by next-generation sequencing (NGS). In addition, we demonstrate the applicability of NGS for the detection of antibiotic resistance markers in plasma.

## Methods

### Study design

Human data are from a secondary analysis of a subset of patients participating in the RAMMSES trial (German Clinical Trials Register, DRKS00000505) [10]. An amendment to the existing approval (Trial-Code-Nr. S123-2009) was written and approved by the local ethics committee.

The observational clinical study was conducted in the surgical intensive care unit (ICU) of Heidelberg University Hospital, Germany. Study and control patients or their legal designees signed a written informed consent. In total, 120 patients in three groups were consecutively enrolled into the study from August 2009 to July 2010. The three groups included: (1) 60 patients with septic shock according to the criteria of the International Sepsis Definitions Conference [11] and due to documented or suspected infection according to the criteria of the International Sepsis Forum Consensus Conference on Definitions of Infection in the Intensive Care Unit [12]; (2) 30 postoperative controls following major abdominal surgery without any evidence of infection; and (3) 30 healthy volunteers. Plasma samples from patients with septic shock were collected at sepsis onset (T0) and 24 h (T1), 4 days (T2), 7 days (T3), 14 days (T4), and 28 days (T5) later. Plasma samples from the postoperative group were collected prior to surgery (T0), immediately following the end of the surgical procedure (T1), and 24 h later (T2). Plasma samples from the volunteer group were collected once (T0).

For this secondary analysis, patients’ electronic medical records were retrospectively screened for results from blood culture testing during septicemia and patients were selected according to availability of microbiological data for similar time points, which led to the smaller sample size within this proof-of-concept study.

### Clinical microbiology

Blood culture testing in Heidelberg University Hospital is routinely performed as described [13]. Whole blood samples are obtained via direct venipuncture (e.g., antecubital vein) applying sterile techniques and 10 mL blood is inoculated to both an aerobic and an anaerobic liquid culture medium (BACTEC PLUS, BD Biosciences, Heidelberg, Germany). Cultures are incubated for 5 days (BACTEC, BD Biosciences, Heidelberg, Germany) and positive cultures are analyzed according to approved in-house hospital standard techniques, including identification by VITEK2 (Biomerieux, Nuertingen, Germany) or MALDI TOF (Bruker, Madison, WI, USA) and automated antimicrobial susceptibility testing (VITEK 2). Quantification of HSV1 DNA and cytomegalovirus DNA from plasma or tracheal secretion was performed via quantitative real time PCR as previously described [14]. Cultivation of wound swabs and catheter and stool samples was carried out as previously described [15, 16].

### Plasma preparation and nucleic acid isolation

Plasma was prepared from blood samples by centrifugation for 10 min at 292 × g and 4 °C, snap frozen, and stored at −80 °C until further processing. Nucleic acids were isolated from thawed plasma after a centrifugation step of 5 min at 1000 × g with the Circulating Nucleic Acid Kit (Qiagen) according to the manufacturer’s protocol with the following exceptions: plasma volumes after centrifugation (from 130 to 790 μl) were adjusted to 1 ml with sterile phosphate buffered saline. Final elution of the nucleic acids from the spin column was carried out with 30 μl molecular biology grade water (5 Prime, Germany). Contamination controls were prepared following the same procedure, starting from 1 ml molecular biology grade water (5 Prime, Germany) and 1 ml of sterile phosphate buffered saline, which was prepared using the Circulating Nucleic Acid Kit (Qiagen). The cfDNA was quantified with the Qubit dsDNA HS Assay Kit (Life Technologies) and quality was assessed by the High Sensitivity DNA Kit on a Bioanalyzer (Agilent).

### Preparation of NGS libraries and sequencing

Libraries for NGS were prepared from 1 ng cfDNA using the Nextera XT library preparation Kit (Illumina) according to the manufacturer’s protocol, with the exception that the final elution after bead clean-up was carried out in 34 μl of resuspension buffer (Illumina). A further contamination control was added by using 5 μl of molecular biology grade water (5 Prime, Germany) as template for the Nextera XT Library Preparation Kit (Illumina). Sequencing of the libraries was performed on a HiSeq2500 (Illumina), resulting in 25–30 million 100-bp single end reads, on average, per sample. Since samples V6, V22, and P6 were initially sequenced with considerably higher numbers of reads, those samples were randomly reduced in silico to 30 million representative subsampled reads.

### Bioinformatics

Raw reads were cleared from potential adapter contamination, quality controlled, and, if necessary, trimmed using BBDuk (https://sourceforge.net/projects/bbmap/). To pass the quality filter, read quality needed to surpass a Phred score of 20 and achieve a minimal length of 50 bp after trimming of low quality and adapter bases. Subsequently, NextGenMap was used to align quality-controlled reads to the human reference genome (hg19) requiring a minimum identity between read and reference genome of 80 %. Reads mapping to the human reference genome and reads with low complexity (consecutive stretches of di- and trinucleotides along the whole read sequence) were excluded from further analysis [17]. Finally, Kraken was used to assign reads to systematic classification using the RefSeq database (release version 68) comprising 35,749 bacterial and 4340 viral genomes complemented by 12 selected fungal genomes (Additional file 1: Table S1). Since several *Xanthomonas* species are described as well-known contaminants, we excluded *Xanthomonas* reads as well as the Illumina sequencing spike-in *PhiX* [18].

To quantitatively compare the number of reads that map to different microbial taxonomic classifications between different samples, we normalized the read counts by the respective library size (Table 1).

**Table 1:** Patient characteristics, cfDNA concentration, and sequencing statistics

ID	Time	Sex	Age (years)	cfDNA (ng/ml plasma)	Sequencing depth	Human reads (%)	Unmapped (%)	Classified (%)
S9	T0	M	82	120.59	30,650,143	92.90	7.10	28.90
S10	T0	M	68	307.83	27,199,593	98.70	1.30	2.85
S11	T0	M	62	805.50	27,073,879	93.61	6.39	20.73
S19	T0	F	62	101.30	26,892,684	98.45	1.55	4.75
S23	T0	M	79	146.70	24,917,032	97.12	2.88	3.85
S26	T0	M	66	1088.90	32,529,889	96.60	3.40	3.24
S60	T0	F	70	70.29	27,381,853	97.10	2.90	4.40
		Average S T0	70	377.30	28,092,153	96.36	3.64	9.82
		Average S all	70	197.23	25,960,730	97.79	2.21	4.24
V5		M	24	35.80	34,203,815	81.90	18.10	12.38
V6		M	29	27.40	30,000,000	98.96	1.04	2.25
V7		F	22	76.40	21,004,601	96.58	3.42	2.35
V13		F	26	23.50	24,449,232	98.09	1.91	3.26
V14		M	28	38.60	37,971,559	97.42	2.58	1.79
V15		M	27	166.80	24,505,696	97.60	2.40	2.88
V16		F	29	70.60	27,220,925	97.06	2.94	2.67
V17		M	26	28.40	20,225,374	98.61	1.39	3.30
V18		M	28	48.80	19,157,938	98.14	1.86	2.46
V19		F	31	33.40	25,776,920	97.08	2.92	2.87
V21		M	22	67.30	25,220,391	97.72	2.28	2.51
V22		M	25	48.20	30,000,000	99.15	0.85	3.25
		Average V	26	55.43	26,644,704	96.52	3.48	3.50
P1	T0	M	58	50.20	22,389,868	96.95	3.05	1.79
P2	T0	M	53	552.00	30,000,000	98.57	1.43	3.35
P3	T0	F	62	109.50	18,796,573	94.69	5.31	1.38
P4	T0	F	72	36.42	28,457,744	94.91	5.09	4.59
P5	T0	M	64	65.38	28,547,804	95.48	4.52	3.72
P7	T0	M	76	82.50	28,845,398	96.69	3.31	1.00
		Average P T0	64	149.33	26,172,898	96.21	3.79	2.64
		Average P T1–T2	64	451.63	25,406,269	97.72	2.28	2.14
		Average P all	64	350.86	25,661,812	97.22	2.78	2.31

Patients were grouped as septic patients (*S*), healthy volunteers (*V*), and non-infected patients following major abdominal surgery (*P*). Of the total reads (sequencing depth), all reads mapped to human reference genome hg19 are classified as human reads; the remaining reads are denoted as unmapped. The proportion of unmapped reads classified to any species using Kraken are specified here as classified. *F* female, *M* male

We introduced the *n* × (*s +* 1) dimensional count matrix D, where *n* is the number of control samples and *s* the number of species detected in all samples. Thus, *D*_*ij*_ defines the number of reads found in control sample *i* for species *j. D*_*i*,(*s* + 1)_ defines the number of reads which cannot be assigned to any species. One notes that *D*_*i*,(*s* + 1)_ is usually larger than the *D*_*ij*_s. Then Eq. 1 is the maximum likelihood estimate of the probability to observe species *j* in a control sample:

1 $$ {\widehat{p}}_j=\frac{{\displaystyle {\sum}_{k=1}^n}{D}_{k,j}}{{\displaystyle {\sum}_{k=1}^n}{D}_{k,s+1}},\ j=1, \dots, s+1 $$

Since the number of reads for one species is typically low, we assumed that the read counts for species *j* are Poisson distributed with parameter:

2 $$ {\lambda}_j=\frac{{\displaystyle {\sum}_{k=1}^n}{D}_{k,j}}{n}. $$

To test this assumption for each species a standard *χ*^2^ goodness of fit test is performed.

For reads sequenced from patient plasma, the same data processing pipeline is applied, which yields a read count vector *C* = (*C*_1_, …, *C*_*s*_, *C*_*s* + 1_). Based on the Poisson distribution with species-specific parameter *λ*_*j*_, the *p* value to observe at least *C*_*j*_ read counts in a patient sample is computed as:

3 $$ P\left(X\ge {C}_j\Big|\ {\lambda}_j\right)={\displaystyle {\sum}_{k\ge {C}_j}\frac{e^{-{\lambda}_j}{\lambda}_j^k}{k!}}. $$

If this *p* value is small, then one would reject the hypothesis that the read count of species *j* in the patient sample follows the Poisson distribution derived from the healthy individuals and conclude that the respective species occurs too often in the patient.

Now, with the given species specific *λ* we can compute the sepsis indicating quantifier (SIQ) score as follows:

4 $$ SI{Q}_j={C}_j\ast -\left(lo{g}_{10}\left(P\left(X\ge {C}_j\Big|{\lambda}_j\right)\right)\right) $$

The SIQ score now gives rise to a quantitative and probabilistic assessment of every detected microbe in the respective sample.

In order to identify resistance genes, potential microbial reads were mapped against the downloaded CARD resistance gene database using NextGenMap with the following parameters: sensitivity (-s) and minimal number of mapped residues (-R) of 0.9. We required 100 % identity between read sequence and target sequence in the CARD database.

## Results

### Elevated levels of cfDNA in septic patients reveal microbial DNA fragments

**Fig. 1:**
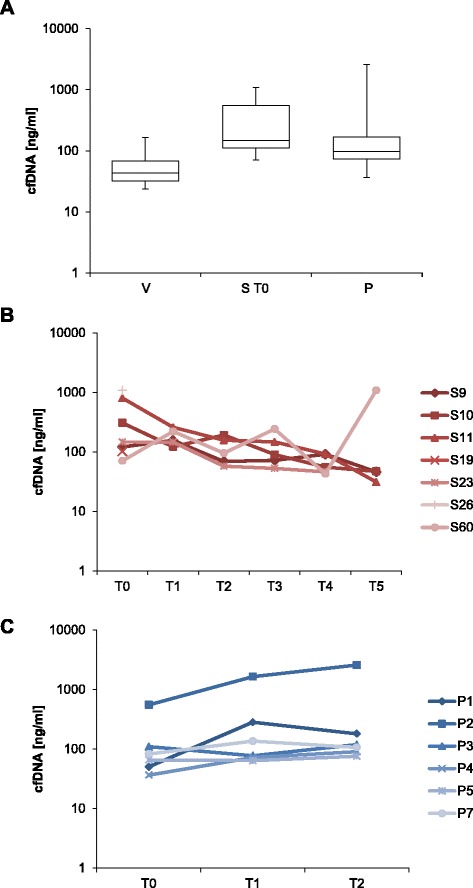
Distribution of cfDNA concentrations over different patient groups and time points. **a** Comparison of cfDNA concentrations between healthy volunteers (*V*), septic patients at the onset of sepsis (*S T0*), and non-infected patients following major abdominal surgery (*P*). **b** Alterations in cfDNA concentrations of septic patients’ plasma samples collected over the observational period of the trial. Samples were obtained at sepsis onset (*T0*), after 24 h (*T1*), 4 days (*T2*), 7 days (*T3*), 14 days (*T4*), and 28 days (*T5*). **c** Comparison of cfDNA concentrations in patients undergoing major abdominal surgery without evidence of infection. Blood samples from the postoperative group were collected prior to surgery (*T0*), immediately following the end of the surgical procedure (*T1*), and 24 h later (*T2*)

In order to test the diagnostic potential of cfDNA to identify infecting microorganisms in septic patients, in total 62 plasma samples from septic patients (S, *n* = 7), healthy volunteers (V, *n* = 12), and patients that underwent abdominal surgery (P, *n* = 6) at different time points were analyzed in this study. Mean ages were 70.5 years, 26.4 years, and 64.2 years for groups S, V, and P, respectively (Table 1). Septic patients were monitored by a comprehensive clinical microbiology diagnostic workup. cfDNA isolation from patient and control plasma revealed a characteristic, predominantly apoptosis-associated size pattern [19, 20] (Additional file 2: Figure S1). Due to the small cohort size, it was not possible to draw statistically relevant conclusions; however, concentrations of cfDNA tend to be increased in patients with septic shock (S, mean 197.23 ng/ml), especially at the onset of sepsis (S T0, mean 377.30 ng/ml) (Fig. 1), which is consistent with other reports [21, 22]. Elevated concentrations were also measured in plasma of post-surgery patients (P T1–T2, mean 451.63 ng/ml) compared with uninfected controls before surgery and healthy volunteers (P T0, mean 149.33 ng/ml; V, mean 55.43 ng/ml). cfDNA sequencing revealed a similar proportion of human reads for all groups with 96.36, 96.52, and 96.21 % in groups S T0, V, and P T0, respectively (Table 1). The average classified reads were higher in septic patients at the onset of sepsis compared with the control groups (9.82, 3.50, and 2.64 % in groups S T0, V, and P T0), indicating a higher bacterial load. It should be noted, however, that the proportion of classified reads includes bleed-through *PhiX* reads and reads classified as from *Xanthomonas campestris*, which were discarded as contaminants in downstream analysis.

### Establishment of the SIQ score as a quantitative score for pathogen calling

**Fig. 2:**
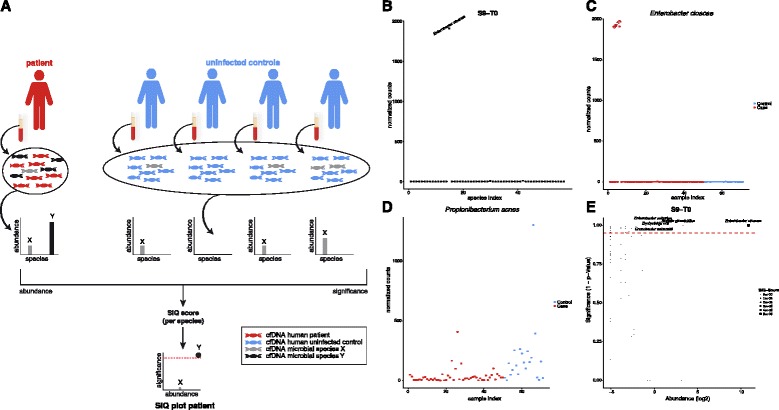
Rationale of the SIQ score and SIQ plot. **a** Outline for obtaining a SIQ score and SIQ plot. Total cfDNA is isolated from a patient’s plasma and sequenced. From sequencing results, human cfDNA are removed after mapping and only unmapped reads are further processed. From these unmapped reads, microbial species are classified and reads are normalized, counted, and sorted by their abundance. For each species obtained from a patient, results are compared with likewise processed samples of uninfected controls, exemplified for microbial species X, which is found in the patient’s sample as well as in most control samples and, therefore, represents a contaminant. However, species Y is found in high abundance only in the patient’s sample and in none of the controls and, therefore, receives a high significance and consequently a high SIQ score, indicated by the radius of its data point in the SIQ plot. **b** Distribution of normalized counts for each species found in the plasma sample of patient S9 at the onset of sepsis (T0). Only the most abundant species, *Enterobacter cloacae*, was labeled. **c** Distribution of the normalized counts for *E. cloacae* for all samples analyzed. *Red*, septic patients; *blue*, controls (elective surgery and healthy volunteers). Only sample S9 with the most abundant *E. cloacae* reads was labeled. **d** Distribution of the normalized counts of *Propionibacterium acnes* for all samples. *Red*, septic patients; *blue*, controls (elective surgery and healthy volunteers). **e** SIQ plot integrating abundance and significance of all species for patient S9 at the onset of sepsis (T0). Coordinates of the data points (species) are the relative abundance (log2) on the *x-axis* and the significance expressed as 1 − *p* value on the *y-axis*. The *dashed line* marks a *p* value of 0.05. Data points with log2 > 0 and *p* value <0.05 are labeled. The SIQ score of a species in the respective sample is integrated as the radius of the data point

Figure 2a summarizes the principle for diagnostic assessment of microbial reads for a patient in comparison with uninfected controls. Normalized read counts of classified, non-human reads for each plasma sample were calculated, resulting in a quantification of distinct species (Additional file 3: Table S6). In case of patient S9 with positive blood cultures for *Enterobacter cloacae* at the onset of sepsis, distinctly higher numbers of *E. cloacae* reads were found compared with a set of species with normalized reads at very low abundances (Fig. 2b). To evaluate the significance of read abundances for all species classified, normalized read counts were compared for each species between all septic patients and controls. The abundance of normalized reads for *E. cloacae* in patients and controls was close to zero except for time point T0 of patient S9 (1,907.33; Fig. 2c; Additional file 4: Figure S2). In contrast, normalized read counts for a common skin commensal like *Propionibacterium acnes* varied significantly between all samples and higher abundances were especially detected in samples obtained from uninfected controls (Fig. 2d; Additional file 5: Figure S3). This effect was observed for several bona fide contaminant species (Additional file 6: Table S2). To support discrimination by relevance, we combined abundance and unlikeliness of observed read counts for each species found in a sample. The log2 ratio of normalized reads is plotted versus 1 − *p* value (Fig. 2e). Species which are significantly represented in a sample with a *p* value <0.05 compared with controls are considered significant (Fig. 2e). An individual SIQ score was calculated as a product of abundance and significance and is indicated by the radius of the corresponding data point. Therefore, the species with highest relevance found in cfDNA of patient S9 at the onset of sepsis was *E. cloacae* with a SIQ score of 586,795 (Additional file 7: Table S3). In contrast, no significant SIQ scores were observed for uninfected postoperative patients (Additional file 7: Table S3 and Additional file 8: Figure S4)

### Clinical relevance of the SIQ score

**Fig. 3:**
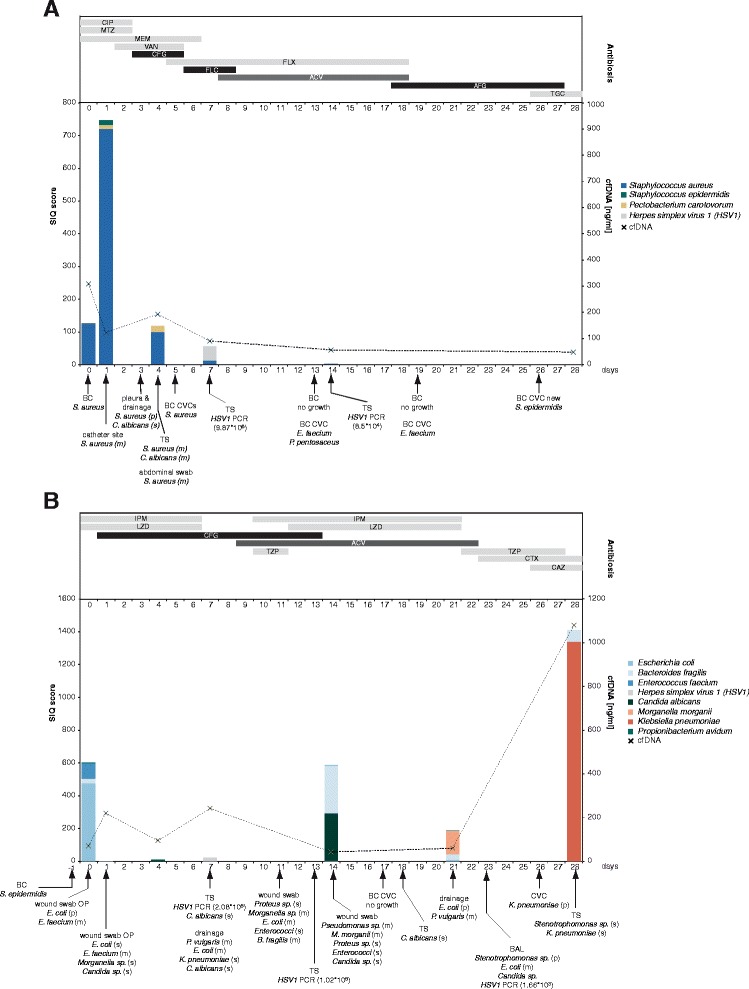
Time course SIQ analyses compared with conventional clinical microbiology data for two patients. **a** Time course of patient S10. A 68-year-old male patient presented with a tumor of his stomach with the need for a gastrectomy. Following the surgical procedure the patient suffered from septic shock due to severe pneumonia without any evidence of an anastomosis insufficiency. *Staphylococcus aureus* was shown to be the dominant organism in different secretions (e.g., tracheal secretion, abdominal wound swab, blood culture, etc.). In addition, pneumonia was shown to be accompanied (respectively boosted) by reactivation of herpes simplex virus type 1 (*HSV1*) in tracheal secretions. Following a prolonged weaning phase, the patient was then able to be discharged to the normal ward 6 weeks after the onset of septic shock. In this figure, the antibiotic treatment regime, SIQ scores for species identified via NGS/SEPseq, and cfDNA concentrations of the respective plasma samples are plotted for the trial period of 28 days. Pertinent (clinical microbiology) laboratory results are marked using *arrows* to indicate the day the clinical specimen was obtained. *Abbreviations*: *BC* blood culture, *CVC* central venous catheter, *TS* tracheal secretion, *HSV* herpes simplex virus, *CIP* ciproflocaxine, *MTZ* metronidazole, *MEM* meropenem, *VAN* vancomycin, *CFG* caspofungin, *FLX* flucloxacillin, *FLC* fluconazole, *ACV* aciclovir, *AFG* anidulafungin, *TGC* tigecycline. Anti-infectives are displayed as antibacterial antibiotics, antimycotics, and antivirals in *light grey*, *black*, and *dark grey*, respectively. The relative amount of bacteria found by conventional clinical microbiology is indicated with plenty (*p*), medium (*m*), or scarce (*s*). (For a detailed list of the anti-infective abbreviations, see Table S5.) **b** Time course of patient S60. Following a complicated course of perforated sigmoid diverticulitis, a 70-year-old female patient presented for reconstruction of bowel continuity. In the postoperative phase the patient developed septic shock due to bowel leakage with the need for surgical revision. Abdominal wound swabs were shown to be positive for *Escherichia coli* and *Enterococcus faecium*. One day later the patient suffered from a second septic hit due to perforation of the colon with the need for surgical colectomy and construction of a stump by Hartmann. Afterwards the patient suffered from another septic hit due to an insufficiency of the stump by Hartmann. Accordingly, one further explorative laparotomy was performed and an intensive abdominal lavage was initiated. In the further course of the septic disease the patient developed a fourth septic hit due to ventilator-associated pneumonia triggered by *E. coli*, *Stenotrophomonas maltophilia*, and *Klebsiella pneumoniae*. Following a prolonged weaning phase the patient was then able to be transferred to the intermediate care ward after 3 months of ICU treatment. Ultimately, the patient could be discharged from hospital another 2 weeks later. Pertinent (clinical microbiology) laboratory results are marked using *arrows* to indicate the day the clinical specimen was obtained. *Abbreviations*: *BC* blood culture, *CVC* central venous catheter, *TS* tracheal secretion, *BAL* bronchoalveolar lavage, *HSV1* herpes simplex virus 1, *IPM* imipenem, *LZD* linezolid, *CFG* caspofungin, *ACV* aciclovir, *TZP* piperacillin-tazobactam, *CTX* cotrimoxazol, *CAZ* ceftazidime. Antibacterial antibiotics are colored in *light grey*. The relative amount of bacteria found by conventional clinical microbiology is indicated with plenty (*p*), medium (*m*), or scarce (*s*). (For a detailed list of the anti-infective abbreviations, see Additional file 9: Table S5)

The SIQ score can also be used for time-course monitoring of a patient’s progression and response to targeted treatment. We therefore compared the SIQ score with clinical microbiology data, supplemented with data on anti-infective therapy (Additional file 9: Table S5) over a trial period of 28 days. Patient S10 developed septic shock due to severe pneumonia following a gastrectomy to remove a tumor of his stomach (Fig. 3a). On study inclusion, blood culture and subsequent samples from different sites were positive for *Staphylococcus aureus*. In addition to *S. aureus*, significant viral burden (herpes simplex virus 1 (HSV1); 9.87 × 10^6^ copies/ml) and low fungal burden (*Candida albicans*) were also detected in other specimens by quantitative PCR (qPCR) or cultivation, respectively. Sequencing of plasma samples confirmed *S. aureus* as the predominant organism in the patient’s blood at all observed time points with SIQ scores of 125.24, 720.41, 12.86, 2, and 0.03. Interestingly, the highest SIQ score for *S. aureus* was observed one day following inclusion and strongly declined afterwards. On day 7 the highest SIQ score was obtained for HSV1 (43.07), which was also detected by qPCR on day 14 in tracheal secretions (8.5 × 10^5^ copies/ml) but otherwise in no other sample (qPCR or NGS). Patient S60 (Fig. 3b) suffered from recurring episodes of bowel leakage accompanied by several septic hits and underwent repetitive reconstructive abdominal surgery. Remarkably, during the course of sepsis, no blood culture was positive for this patient, with the exception of a single bottle positive for *Staphylococcus epidermidis* one day before study inclusion. However, wound swabs and intraabdominal lavage were positive for *Escherichia coli* and *Enterococcus faecium.* Tracheal secretions furthermore tested positive for HSV1 and/or *C. albicans*. During the course of infection, the patient developed a ventilator-associated pneumonia which was due to *E. coli*, *Stenotrophomonas maltophilia*, and *Klebsiella pneumoniae*. In contrast to standard blood culture, SIQ score analyses of this patient’s plasma were in good agreement with non-blood culture clinical microbiology results over the complete time course. Samples collected at the early septic phase (days 0, 1, 4, 7, 14, and 21) confirmed the clinical representation of a polymicrobial abdominal infection due to *E. coli*, *E. faecium*, and *Bacteroides fragilis.* Furthermore, results from qPCR of HSV1 from tracheal secretions could be confirmed by NGS (day 7). The progression of the patient towards ventilator-associated pneumonia at the end of our study period was also confirmed by SIQ scores of 1341.37 for *K. pneumonia* and 68.58 for *B. fragilis* in plasma obtained on day 28. For additional patients NGS results from plasma samples matched data from other specimens, such as tracheal secretion, swabs, or catheter cultivation (Additional files 10, 11, and 12: Figures S5–S7).

### Identification of resistance genes

**Fig. 4:**
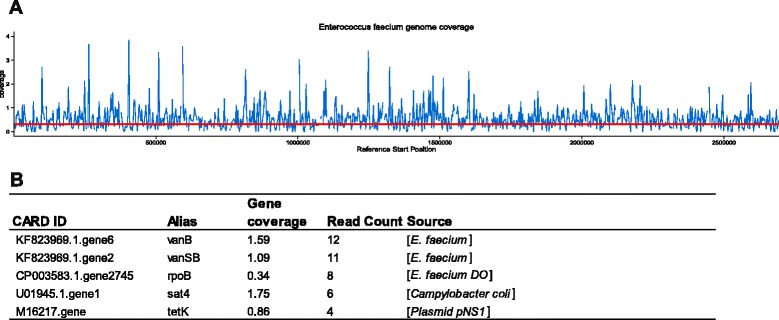
Genome coverage and resistance profile of a patient infected with vancomycin-resistant *E. faecium* (VRE). **a** Mean genome coverage of approximately 0.3 of the *E. faecium* genome (2.8 Mb). **b** Table with hits to the CARD database. The CARD/GenBank accession number is listed as well as the alias gene name, gene coverage calculated from read length ratio to gene length, number of reads mapped to this gene, and the respective organism to which the sequence is assigned

Resistance to antibiotics is mediated by the expression of resistance genes, including *mecA* or *vanA/B* mediating resistance to methicillin or vancomycin in Gram-positive cocci, for example. We analyzed one plasma sample from a 59-year-old woman that suffered from sepsis following liver transplantation. Sequencing the corresponding plasma cfDNA at high depth (Additional file 13: Table S4) showed even coverage of the *E. faecium* genome (Fig. 4a; Additional file 14: Figure S9) and revealed unambiguous hits for resistance genes in the CARD database, including *vanB*, *vanS*_*B*_, *tet1*, and *sat4* (Fig. 4b). Remarkably, our findings match with microbiological data showing that the infectious organism was *E. faecium* exhibiting vancomycin and tetracycline resistance (VRE), among others. Thus, assuming a sufficient sequence coverage, identification of genes conferring resistance using an unbiased high-throughput sequencing approach seems to be possible in principle.

## Discussion

In recent years, several reports have been published on the use of metagenomics for the identification of viruses or microbes in patient samples. Most of these reports describe the identification of different viruses, including influenza A, norovirus, Ebola, arenaviruses, or emerging viruses from various specimens like nasopharyngeal swabs, serum, or solid tissue [23–29]. In addition, metagenomics, amongst other methods, has also been applied for identification of bacteria from urine, vaginal swabs, or sputum [30–33]. However, to the best of our knowledge, we report here the first proof-of-concept for quantitative NGS-based diagnosis of septicemia using plasma samples from septic patients and uninfected controls based on free circulating DNA. Several attempts have been made to use cfDNA quantification as a diagnostic and prognostic marker for sepsis outcome [34–36]. However, elevated cfDNA levels resulting from apoptotic or necrotic processes have also been described in other physiologic or pathologic conditions, including pregnancy, exercise, inflammatory diseases, trauma due to stroke, cancer, and surgery. Consequently, it is controversial whether elevated levels of circulating cfDNA are of diagnostic and prognostic value per se [36, 37]. Five out of seven septic patients (S9, S10, S19, S23, S60) underwent major abdominal surgery within 2 days of sepsis onset, which likely contributed to elevated cfDNA concentrations. However, septic patients showed a significantly higher share of cfDNA that could be assigned to microbial origin, suggesting a direct release of DNA from bacteria or co-release from phagocytic cells. This cfDNA could originate from both live and dead pathogens. However, as cfDNA is subject to considerable turnover, with a half-life in the range of minutes [38, 39], and obtained SIQ scores are very dynamic during the time course of infection, this suggests that cfDNA levels from microbial origin seem to precisely reflect the dynamics of infection, irrespective of whether cfDNA is released by dead or living cells. Due to the very short half-life of cfDNA, we therefore would also not expect to face a significant false positive rate. However, as our study only comprises a limited number of cases, further research and more comprehensive cohorts are needed to elucidate the source and mechanism of cfDNA release and its impact on false positive rates.

In patients suffering from severe sepsis or septic shock, positive blood cultures are obtained in only a fraction of cases despite a proven underlying rate of bacterial infection of 33 % [40–42]. This is partially attributable to technical shortfalls in blood culture acquisition but is also due to fastidious organisms or very low rates of viable microorganisms in the blood stream [43]. A molecular approach with higher sensitivity for sepsis might, therefore, overcome the aforementioned limitations of classic microbiological diagnostics. However, state of the art molecular approaches based on PCR amplification of target sequences suffer from limited power to discriminate between contaminations, colonization, and infections, often revealing ambiguous or invalid results. In this context, NGS-based diagnostic testing might offer several advantages over PCR assays: (i) NGS is an open platform, providing the opportunity to detect bacterial, fungal, and viral pathogens in a single assay; (ii) NGS is quantitative through counting of sequence reads and calculation of statistical significance; (iii) NGS is unbiased and untargeted so benefits from any DNA sequence information within patient specimens, potentially delivering higher sensitivity and specificity. However, although NGS is becoming increasingly important in clinical microbiology (e.g., for strain typing), only sporadic reports of NGS-analyzed clinical specimens have been published to date, including one actionable single-case report of NGS-based diagnosis of *Leptospira* from cerebrospinal fluid [44–49]. Another recent paper, by De Vlaminck and coworkers [50], reports the identification of predominantly viral pathogens in cfDNA from patients after lung transplantation. Nevertheless, we currently lack NGS-based diagnostic approaches that, by combining quantitative values with statistical significance, provide a measure for relevance. Especially in specimens like plasma, where only the lowest amounts of microbial DNA can be expected, the specificity is challenged by the detection of microbial contaminants from laboratory reagents, workflows, or specimen sampling [18, 51]. We therefore developed a SIQ score to discriminate signal reads from noise caused by contaminant or commensal species. Since the incidence of viral and fungal infections in sepsis is less frequent, the SIQ scores for fungi and viruses will generally be higher than those for bacteria (as seen in patient S23; Additional file 11: Figure S6) as they are also less often detected in control samples. Consequently, the robustness of the SIQ score will be increased by using the most complete database of relevant genomes and a control group large enough to capture even small variances in the microbiome of healthy individuals.

Using our current workflow, a good correlation of NGS results with blood culture and other patient specimens was observed for all patients except patient S60. Moreover, this agreement was observed not only for bacteria but also for viruses and fungi. For patient S60 we found *E. coli* and *E. faecium* in plasma, which is in good agreement with wound swab findings but in contrast to blood culture, where *S. epidermidis* was identified one day before. Clinical records suggest that this patient suffered from gut complications, indicating that the NGS-based approach might be more sensitive and specific than blood culture, which has to be systematically evaluated by further studies.

Importantly, we could also demonstrate the potential of quantitative NGS-based diagnosis to identify genes conferring antibiotic resistance in plasma cfDNA. We unambiguously identified from a septic patient that underwent liver transplantation a number of reads that match with 100 % identity to *vanB* and *vanS*_*B*_, known to confer resistance to vancomycin. Indeed, we could identify VRE as the infecting strain as confirmed by microbiological testing. Meanwhile, we were also able to detect *mecA* from methicillin-resistant *S. aureus* in other patient plasma samples (data not shown), indicating that the identification of major resistance genes is feasible with our approach. However, the applicability of the CARD antibiotic resistance database for diagnostic purposes is currently limited to genes which, per se, confer resistance to antibiotics, whereas the heterogeneity of resistance mechanisms also comprises point mutations, gene expression changes, and posttranslational modifications, for example. Consequently, using the current setup of the database and workflows, it is possible to identify resistance mechanisms based on acquisition of specific resistance genes like *mecA*, *vanA/B*, KPC, etc. but not necessarily to detect all resistance mechanisms comprehensively. Furthermore, it should be noted that mapping of reads to resistance-related genes relies on high read coverage for the respective species. For routine detection of resistance genes, enrichment strategies and complementation with expression data might be helpful in elucidating additional resistance mechanisms.

Finally, using our workflow to quantitatively identify microbial and viral nucleic acids in patient plasma (Additional file 15: Figure S8), the current total turnaround time starting from plasma adds up to approximately 31 h, where the most time-consuming step is sequencing, which was accomplished in rapid run mode on a HiSeq2500 within 16 h. This is competitive with standard blood culture with a time to positivity of 24–120 h, where automated blood culture systems are routinely incubated for 5 days [43]. However, recent developments in sequencing technologies (i.e., Oxford Nanopore’s MinION device) indicate that it will be possible to sequence even larger amounts of DNA in shorter times in a bedside setup. Using this technology it has already been reported that strain identification of clinically relevant strains and the corresponding resistance profiles is possible in approximately 2 h [52]. Furthermore, the identification of resistance genes has been shown to be manageable in 2 to 12 h, which makes a “same day” diagnosis possible [52]. Nanopore sequencing would, therefore, allow a diagnostic decision within 3–4 h after initial diagnosis of sepsis using our workflow, providing a reasonable timeframe for effective therapeutic intervention. It should also be mentioned that current sequencing costs are of considerable importance for diagnostics. Although costs per base are constantly dropping, diagnostic approaches based on NGS are still comparatively expensive. Therefore, we expect that such a diagnostic workflow will be in a cost range similar to that of noninvasive prenatal testing (NIPT) when it was introduced several years before. This might limit a corresponding approach for diagnosis in critically ill patients in the beginning, for whom overall costs per patient and day are relatively high. But as seen for NIPT, costs per assay might significantly drop in short order following a broader application of NGS in clinics, thus making such an approach conceivable also from a financial point of view.

## Conclusions

This work shows for the first time that identification of viruses, bacteria, and fungi from cfDNA in plasma of septic patients not only is technically feasible but also provides a basis to differentiate the relevant infecting organisms by establishing a quantitative score. These characteristics go beyond state-of-the-art molecular approaches for the diagnosis of infecting organisms in septic specimens, which are not open but instead based on PCR amplification of defined targets and are, in most cases, just qualitative in nature. Therefore, this approach allows for an unbiased analysis of bloodstream infections, which might be especially useful for the diagnosis of cases where classic microbiological or molecular diagnostic approaches fail. However, although we think that our approach might be more sensitive and specific than state-of-the-art technologies, additional clinical trials will be needed to exactly define the sensitivity and specificity as well as the positive and negative predictive value as this case study was limited by the low number of patients.

## Abbreviations

cfDNA, cell-free DNA; HSV, herpes simplex virus; NGS, next generation sequencing; qPCR, quantitative PCR; SIQ, sepsis indicating quantifier; VRE, vancomycin-resistant *E. faecium*.

## Additional files

Additional file 1: Table S1. List of fungal reference genomes included in the kraken database to identify microbial reads in non-human reads. (XLSX 11 kb) Additional file 2: Figure S1. Bioanalyzer profiles of cfDNA isolated from septic patients, non-infected (post-)surgery controls, and healthy volunteers. **a** Gel-like visualization of the cfDNA profiles run on the Agilent Bioanalyzer with a High Sensitivity DNA chip. **b** Corresponding electropherograms, where the two outermost peaks are internal size standards. (PDF 661 kb) Additional file 3: Table S6. Total read counts per sample and species. (XLSX 57 kb) Additional file 4: Figure S2. Distribution of species-specific normalized read counts in septic patients and controls for major pathogens in the sepsis setting. Red: septic patients, blue: controls (elective surgery (timepoint T0) and healthy volunteers). (PDF 19 kb) Additional file 5: Figure S3. Distribution of species-specific normalized read counts in septic patients and controls for potential contaminant species. *Red*, septic patients; *blue*, controls (elective surgery (timepoint T0) and healthy volunteers). (PDF 18 kb) Additional file 6: Table S2. List of contaminant species identified from water controls. (XLSX 10 kb) Additional file 7: Table S3. Patient characteristics, sequencing statistics, including normalized germ read counts, and SIQ scores for the complete dataset. (XLSX 21 kb) Additional file 8: Figure S4. SIQ plot for post-surgery patients P1 T1 and P2 T1. (PDF 72 kb) Additional file 9: Table S5. Anti-infective abbreviations. (XLSX 9 kb) Additional file 10: Figure S5. Time course of patient S9. An 82-year-old male patient presented with a tumor of his bile duct with the need for an enlarged right-sided hemihepatectomy. Following the surgical procedure, the patient suffered from septic shock due to Ogilvie syndrome and a right-sided hemicolectomie had to be performed. Septic shock was paralleled by repetitive positive blood cultures with *Enterobacter cloacae*, which was shown to be the cause of ventilator-associated pneumonia 1 week after sepsis onset. Following antibiotic treatment, two different biotypes of *E. cloacae* could be observed, both fulfilling the criteria of being multidrug resistant. The patient died 9 weeks after the onset of septic shock. In this figure, the antibiotic treatment regime, SIQ scores for species identified via NGS, and cfDNA concentrations of the respective plasma samples are plotted over the timeline of the trial period for patient S9. Pertinent clinical microbiology laboratory results are marked using *arrows* to indicate the day the clinical specimen was obtained. *Abbreviations*: *BC* blood culture, *CVC* central venous catheter, *TS* tracheal secretion, *NL* non-lysing, *CIP* ciproflocaxine, *MTZ* metronidazole, *IPM* imipenem, *LZD* linezolid, *FLC* fluconazole, *VAN* vancomycin. Anti-infectives are displayed as antibacterial antibiotics, antimycotics, and antivirals in *light grey*, *black*, and *dark grey*, respectively. The relative amount of bacteria found by conventional clinical microbiology is indicated with plenty (*p*), medium (*m*), or scarce (*s*). (For a detailed list of the anti-infective abbreviations, see Additional file 9: Table S5.) (PDF 16 kb) Additional file 11: Figure S6. Time course of patient S11. A 62-year-old male patient presented with a multilocular hepatocellular carcinoma with the need for a left-sided hemihepatectomy. Following the surgical procedure the patient suffered from septic shock due to severe pneumonia with *Klebsiella pneumoniae* as the dominant organism in blood cultures as well as tracheal secretions. Empiric antibiotic therapy was performed with imipenem, which was then switched to moxifloxacin based on the susceptibility findings. In the further course of the disease, *K. pneumoniae* was shown to be multidrug resistant. Although antibiotic therapy was adapted according to the findings of susceptibility testing, the pulmonary septic focus could not be removed sufficiently. In the end, the patient died from ongoing septic shock due to pneumonia with *K. pneumonia* 2 months after study inclusion. In addition, septic disease was shown to be accompanied by a reactivation of herpes simplex virus type 1 (*HSV1*) as well as cytomegalovirus (*CMV*) in different secretions as assessed by a PCR-based diagnostic procedure. These findings were in good agreement with next generation sequencing (NGS) of plasma. In this figure, the antibiotic treatment regime, SIQ scores for species identified via NGS, and cfDNA concentrations of the respective plasma samples are plotted over the timeline of the trial period for patient S11. Pertinent (clinical microbiology) laboratory results are marked using *arrows* to indicate the day the clinical specimen was obtained. *Abbreviations*: *BC* blood culture, *CVC* central venous catheter, *TS* tracheal secretion, *GNST* Gram-negative staphylococci, *HSV1* herpes simplex virus 1, *IPM* imipenem, *VAN* vancomycin, *MXF* moxiflocaxin, *CIP* ciprofloxacin, *TGC* tigecycline, *CAZ* ceftazidime. Antibacterial antibiotics are displayed in *light grey*. The relative amount of bacteria found by conventional clinical microbiology is indicated with plenty (*p*), medium (*m*), or scarce (*s*). (For a detailed list of the anti-infective abbreviations, see Additional file 9: Table S5) (PDF 16 kb) Additional file 12: Figure S7. Time course of patient S23. A 77-year-old male patient presented with septic shock due to an acute abdominal infection. An ischemic colitis with a perforation of the sigma and severe peritonitis was identified to be the septic focus, so the patient underwent surgical colectomy. Abdominal wound swabs as well as the corresponding blood cultures were shown to be positive for *Enterococcus faecium* 2 days before inclusion in the study cohort. Empiric antibiotic therapy with imipenem and linezolid was therefore proven to be appropriate. In addition, this patient also revealed a reactivation of herpes simplex virus type 1 (*HSV1*) in tracheal secretions. These PCR-based findings could also be confirmed by next generation sequencing (*NGS*) of plasma. The antibiotic treatment regime, SIQ scores for species identified via NGS, and cfDNA concentrations of the respective plasma samples are plotted over the timeline of the trial period for patient S23. Pertinent (clinical microbiology) laboratory results are marked using *arrows* to indicate the day the clinical specimen was obtained. *Abbreviations*: *BC* blood culture, *TS* tracheal secretion, *CoNST* coagulase negative staphylococci, *IMP* imipeneme, *LZD* linezolid, *TZP* tazobactam, *CLI* clindamycin, *FLC* fluconazole. Anti-infectives are colored as antibacterial antibiotics and antimycotics in *light grey* and *black*, respectively. The relative amount of bacteria found by conventional clinical microbiology is indicated with plenty (*p*), medium (*m*), or scarce (*s*). (For a detailed list of the anti-infective abbreviations, see Additional file 9: Table S5.) (PDF 15 kb) Additional file 13: Table S4. Sequencing characteristics of the resistance-characterized patient sample. (XLSX 8 kb) Additional file 14: Figure S9. Mapping of *Enterococcus faecium* identified reads on the genome of *Enterococcus faecium DO* (NC_017960.1) using different minimum identity values while mapping. **a** Minimum identity of 65 %; **b** minimum identity of 70 %; **c** minimum identity of 85 %; **d** minimum identity of 90 %; **e** minimum identity of 95 %; **f** minimum identity of 100 %. (PDF 382 kb) Additional file 15: Figure S8. Workflow and time distribution of NGS-based pathogen identification and blood culture. Flow chart and bar chart of the individual steps and the time required for the NGS-based identification of bacteremia-causing species and conventional blood culture. As time to positivity varies substantially, a time frame of 24 to 120 h is given for blood culture. (PDF 42 kb)
