# Supplementary figures and images for: Next-generation sequencing diagnostics of bacteremia in septic patients
Source: Genome Med. 2016 Jul 1;8:73. doi: 10.1186/s13073-016-0326-8 (PMC4930583; doi:10.1186/s13073-016-0326-8)

**A**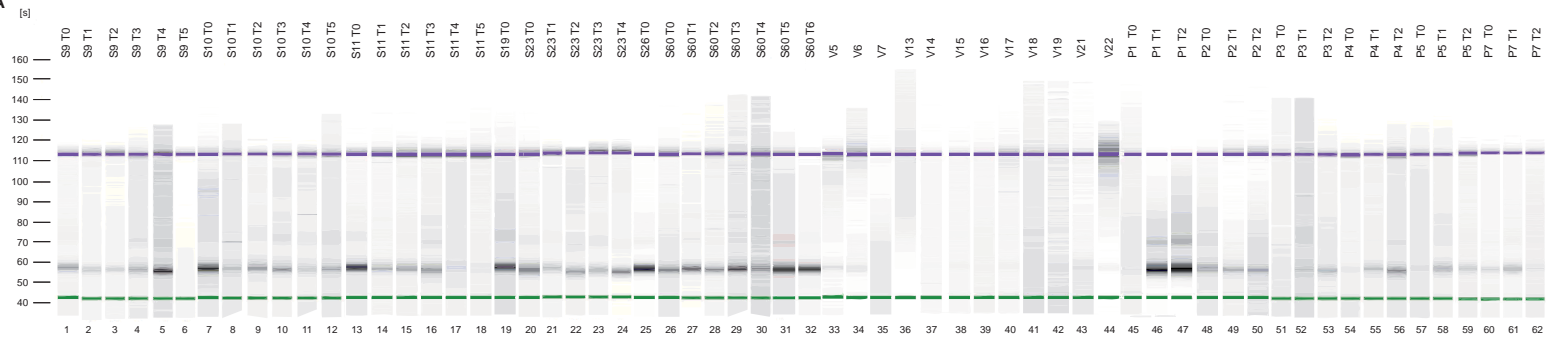**B**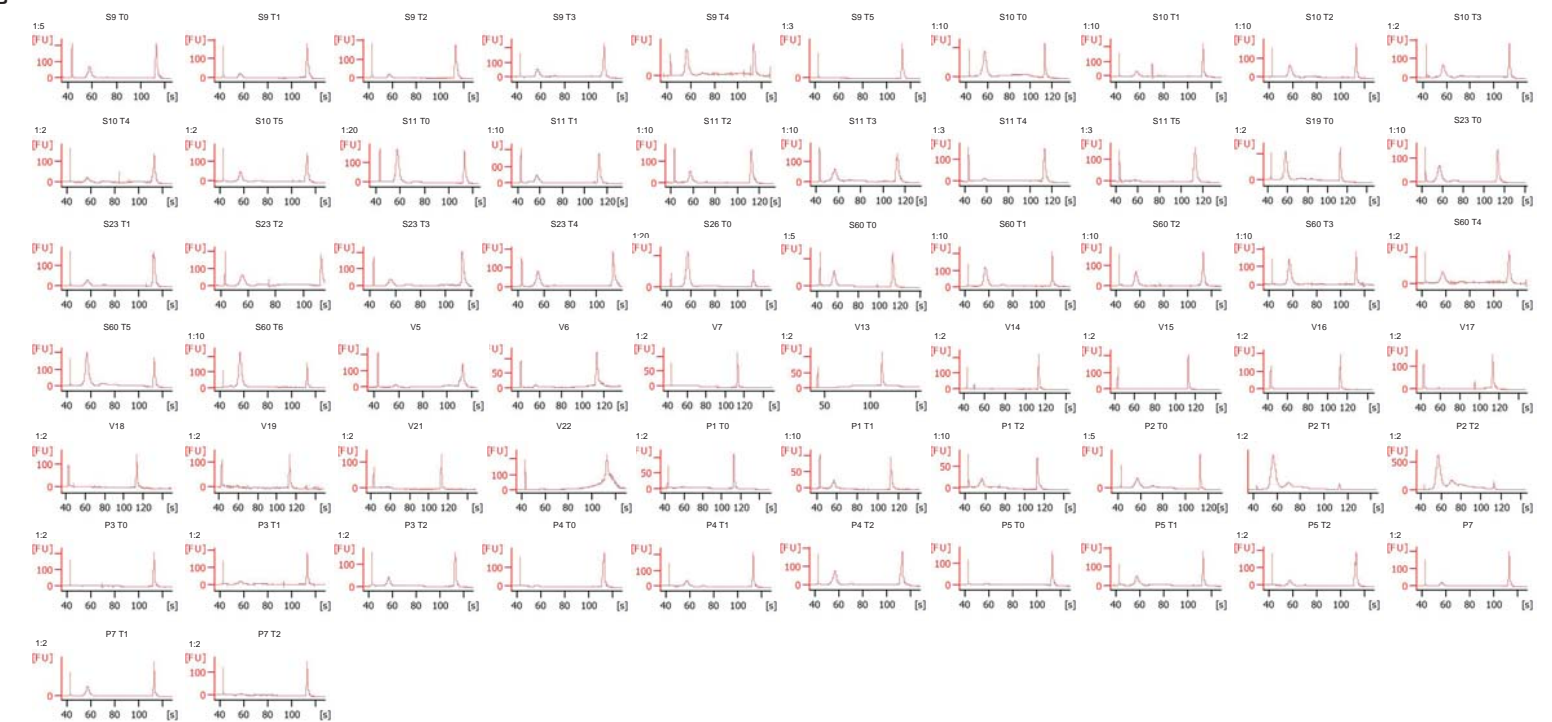

Supplement: Additional file 2: Figure S1. — Bioanalyzer profiles of cfDNA isolated from septic patients, non-infected (post-)surgery controls, and healthy volunteers. a Gel-like visualization of the cfDNA profiles run on the Agilent Bioanalyzer with a High Sensitivity DNA chip. b Corresponding electropherograms, where the two outermost peaks are internal size standards. (PDF 661 kb) [file 13073_2016_326_MOESM2_ESM.pdf]

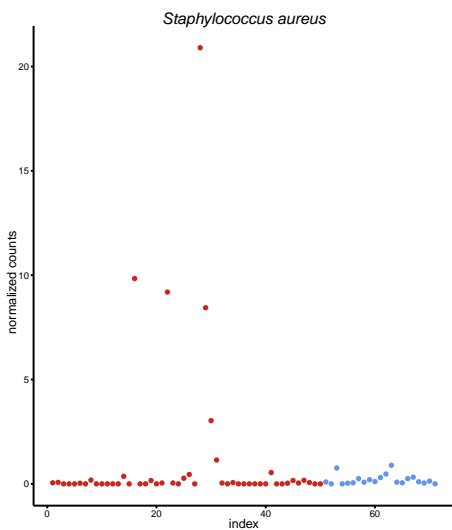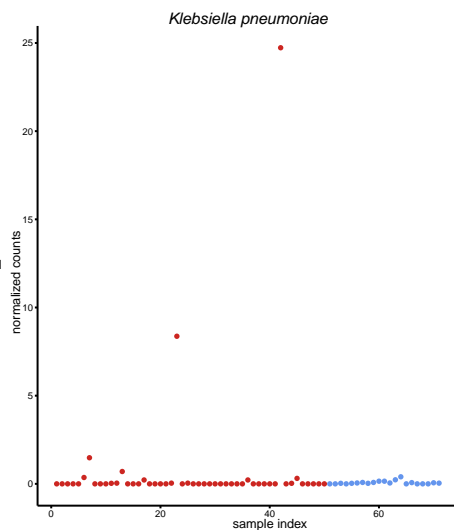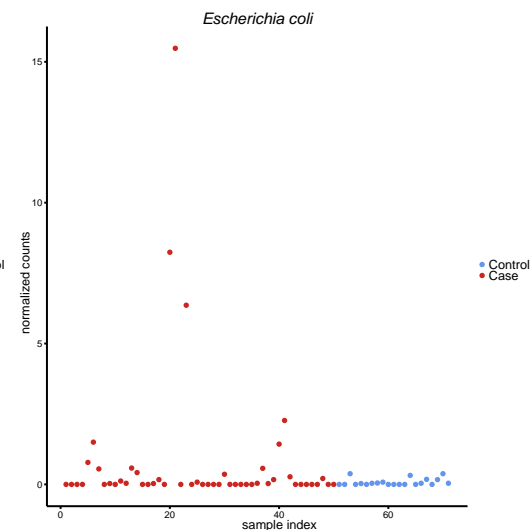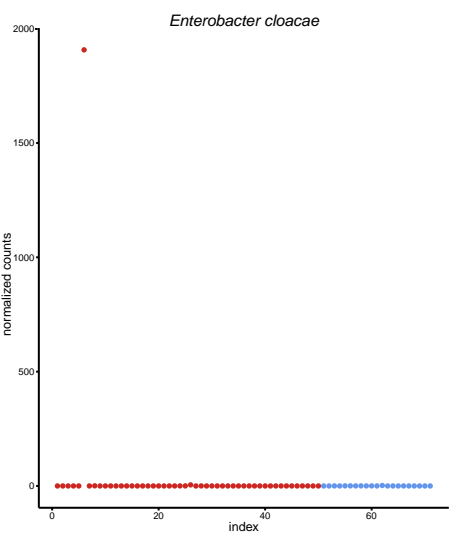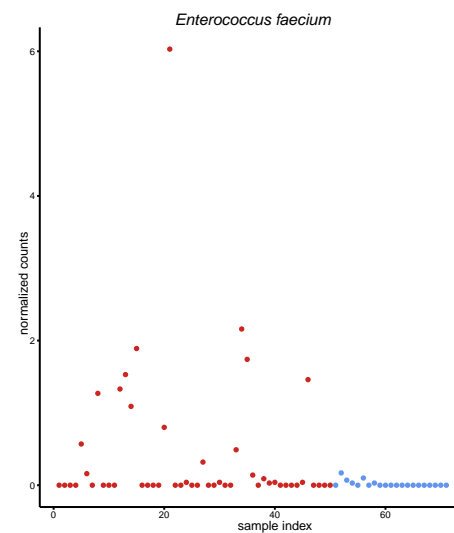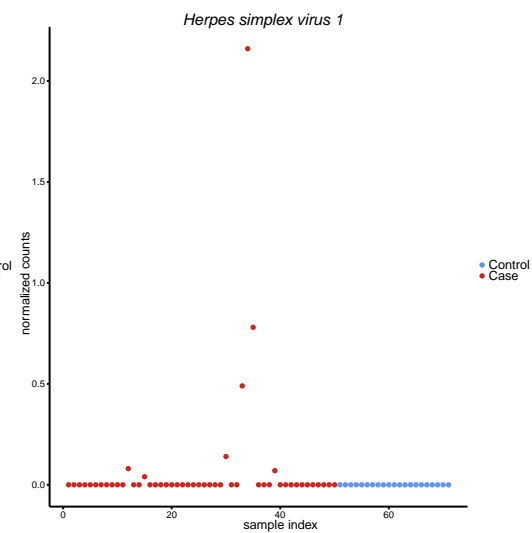

Supplement: Additional file 4: Figure S2. — Distribution of species-specific normalized read counts in septic patients and controls for major pathogens in the sepsis setting. Red: septic patients, blue: controls (elective surgery (timepoint T0) and healthy volunteers). (PDF 19 kb) [file 13073_2016_326_MOESM4_ESM.pdf]

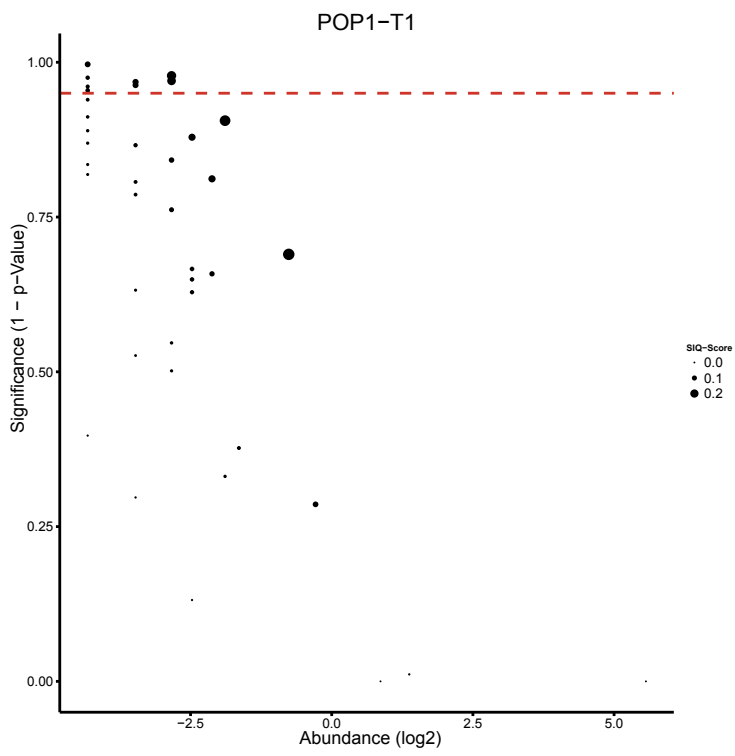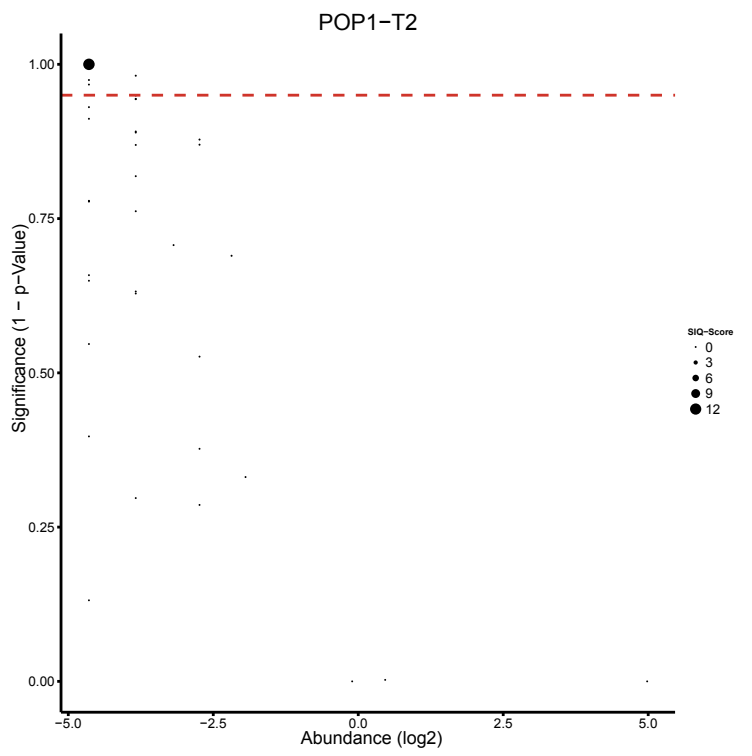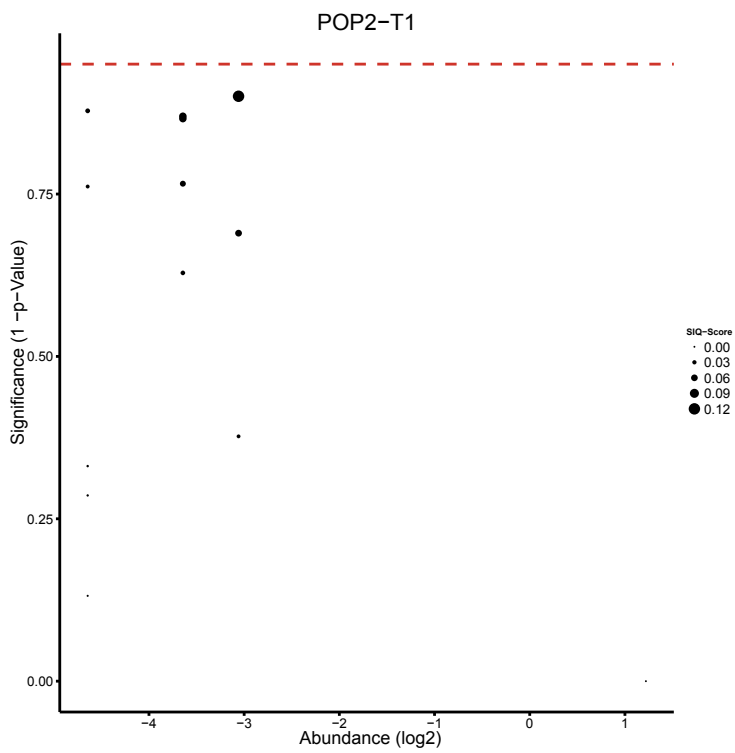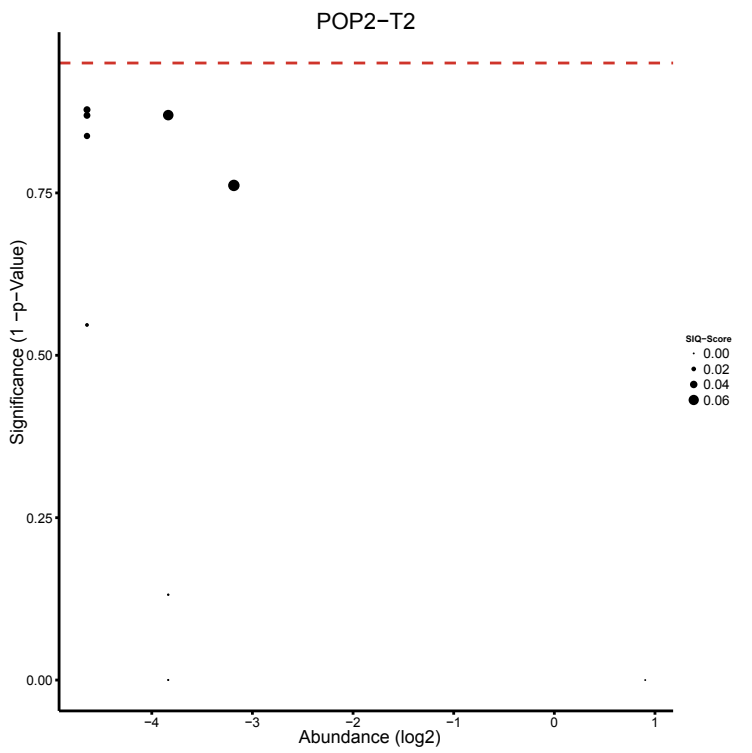

Supplement: Additional file 8: Figure S4. — SIQ plot for post-surgery patients P1 T1 and P2 T1. (PDF 72 kb) [file 13073_2016_326_MOESM8_ESM.pdf]

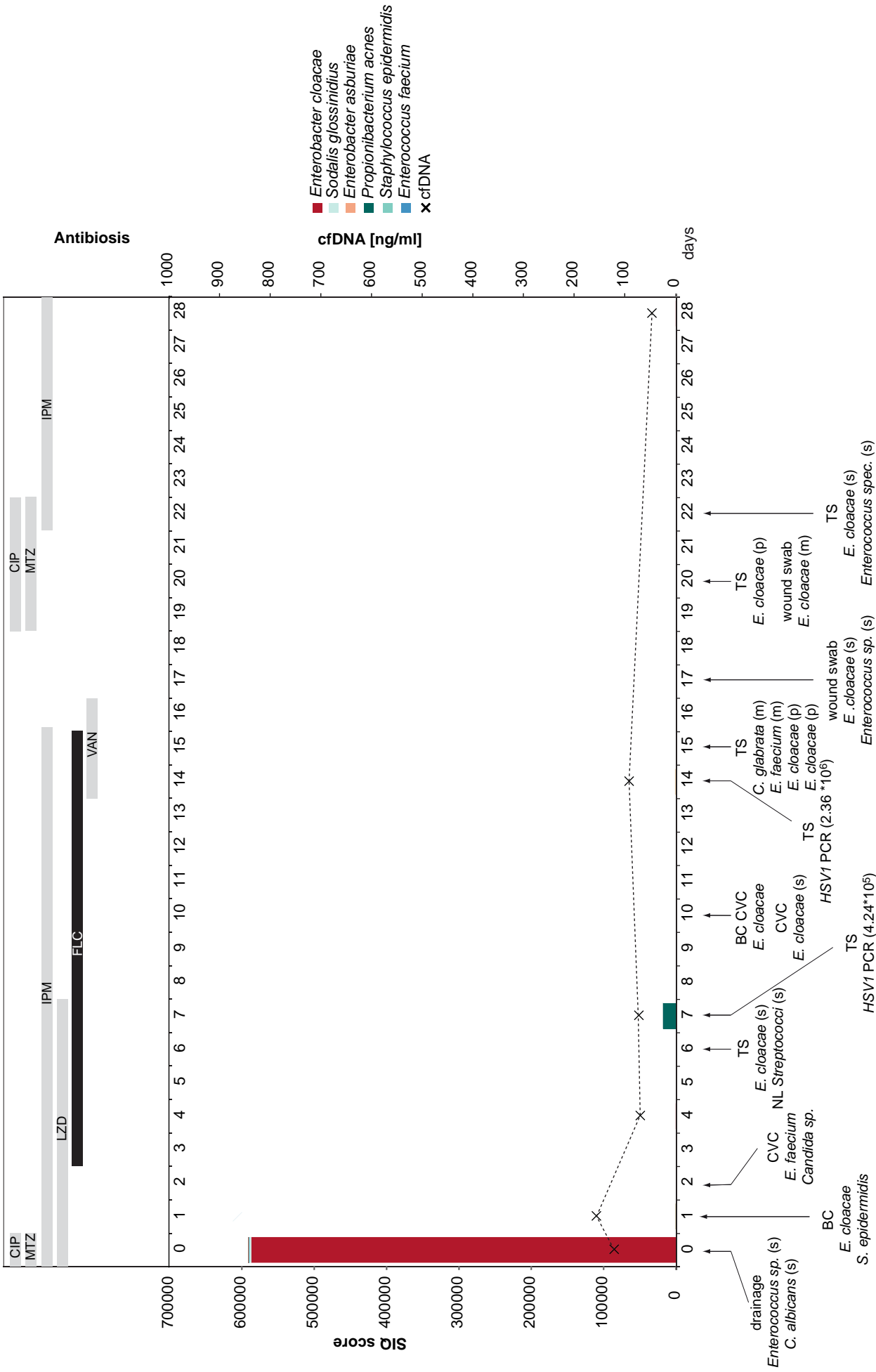

Supplement: Additional file 10: Figure S5. — Time course of patient S9. An 82-year-old male patient presented with a tumor of his bile duct with the need for an enlarged right-sided hemihepatectomy. Following the surgical procedure, the patient suffered from septic shock due to Ogilvie syndrome and a right-sided hemicolectomie had to be performed. Septic shock was paralleled by repetitive positive blood cultures with Enterobacter cloacae, which was shown to be the cause of ventilator-associated pneumonia 1 week after sepsis onset. Following antibiotic treatment, two different biotypes of E. cloacae could be observed, both fulfilling the criteria of being multidrug resistant. The patient died 9 weeks after the onset of septic shock. In this figure, the antibiotic treatment regime, SIQ scores for species identified via NGS, and cfDNA concentrations of the respective plasma samples are plotted over the timeline of the trial period for patient S9. Pertinent clinical microbiology laboratory results are marked using arrows to indicate the day the clinical specimen was obtained. Abbreviations: BC blood culture, CVC central venous catheter, TS tracheal secretion, NL non-lysing, CIP ciproflocaxine, MTZ metronidazole, IPM imipenem, LZD linezolid, FLC fluconazole, VAN vancomycin. Anti-infectives are displayed as antibacterial antibiotics, antimycotics, and antivirals in light grey, black, and dark grey, respectively. The relative amount of bacteria found by conventional clinical microbiology is indicated with plenty (p), medium (m), or scarce (s). (For a detailed list of the anti-infective abbreviations, see Additional file 9: Table S5.) (PDF 16 kb) [file 13073_2016_326_MOESM10_ESM.pdf]

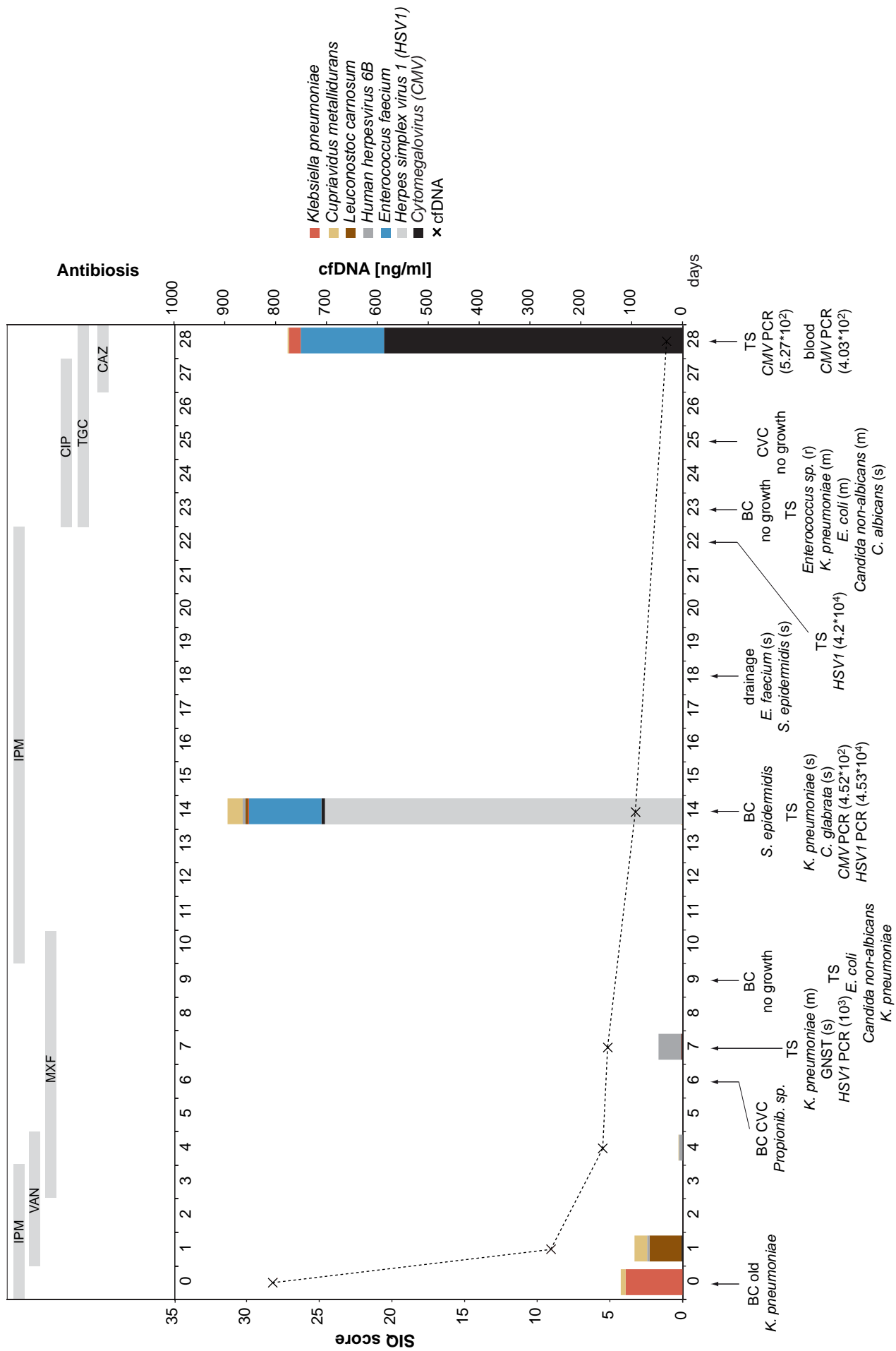

Supplement: Additional file 11: Figure S6. — Time course of patient S11. A 62-year-old male patient presented with a multilocular hepatocellular carcinoma with the need for a left-sided hemihepatectomy. Following the surgical procedure the patient suffered from septic shock due to severe pneumonia with Klebsiella pneumoniae as the dominant organism in blood cultures as well as tracheal secretions. Empiric antibiotic therapy was performed with imipenem, which was then switched to moxifloxacin based on the susceptibility findings. In the further course of the disease, K. pneumoniae was shown to be multidrug resistant. Although antibiotic therapy was adapted according to the findings of susceptibility testing, the pulmonary septic focus could not be removed sufficiently. In the end, the patient died from ongoing septic shock due to pneumonia with K. pneumonia 2 months after study inclusion. In addition, septic disease was shown to be accompanied by a reactivation of herpes simplex virus type 1 (HSV1) as well as cytomegalovirus (CMV) in different secretions as assessed by a PCR-based diagnostic procedure. These findings were in good agreement with next generation sequencing (NGS) of plasma. In this figure, the antibiotic treatment regime, SIQ scores for species identified via NGS, and cfDNA concentrations of the respective plasma samples are plotted over the timeline of the trial period for patient S11. Pertinent (clinical microbiology) laboratory results are marked using arrows to indicate the day the clinical specimen was obtained. Abbreviations: BC blood culture, CVC central venous catheter, TS tracheal secretion, GNST Gram-negative staphylococci, HSV1 herpes simplex virus 1, IPM imipenem, VAN vancomycin, MXF moxiflocaxin, CIP ciprofloxacin, TGC tigecycline, CAZ ceftazidime. Antibacterial antibiotics are displayed in light grey. The relative amount of bacteria found by conventional clinical microbiology is indicated with plenty (p), medium (m), or scarce (s). (For a detailed list of the anti-infective abbreviat [file 13073_2016_326_MOESM11_ESM.pdf]

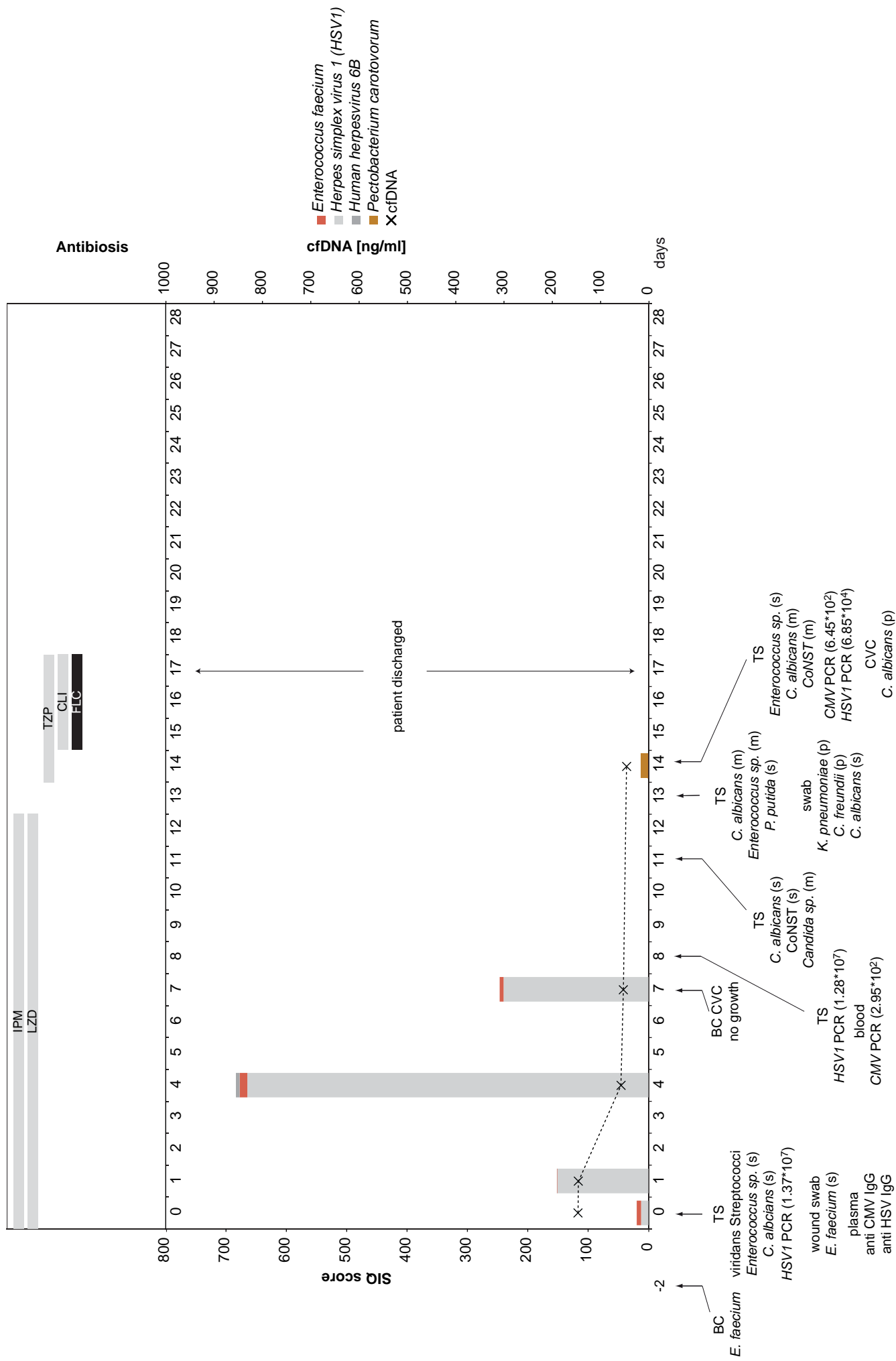

Supplement: Additional file 12: Figure S7. — Time course of patient S23. A 77-year-old male patient presented with septic shock due to an acute abdominal infection. An ischemic colitis with a perforation of the sigma and severe peritonitis was identified to be the septic focus, so the patient underwent surgical colectomy. Abdominal wound swabs as well as the corresponding blood cultures were shown to be positive for Enterococcus faecium 2 days before inclusion in the study cohort. Empiric antibiotic therapy with imipenem and linezolid was therefore proven to be appropriate. In addition, this patient also revealed a reactivation of herpes simplex virus type 1 (HSV1) in tracheal secretions. These PCR-based findings could also be confirmed by next generation sequencing (NGS) of plasma. The antibiotic treatment regime, SIQ scores for species identified via NGS, and cfDNA concentrations of the respective plasma samples are plotted over the timeline of the trial period for patient S23. Pertinent (clinical microbiology) laboratory results are marked using arrows to indicate the day the clinical specimen was obtained. Abbreviations: BC blood culture, TS tracheal secretion, CoNST coagulase negative staphylococci, IMP imipeneme, LZD linezolid, TZP tazobactam, CLI clindamycin, FLC fluconazole. Anti-infectives are colored as antibacterial antibiotics and antimycotics in light grey and black, respectively. The relative amount of bacteria found by conventional clinical microbiology is indicated with plenty (p), medium (m), or scarce (s). (For a detailed list of the anti-infective abbreviations, see Additional file 9: Table S5.) (PDF 15 kb) [file 13073_2016_326_MOESM12_ESM.pdf]

**A**

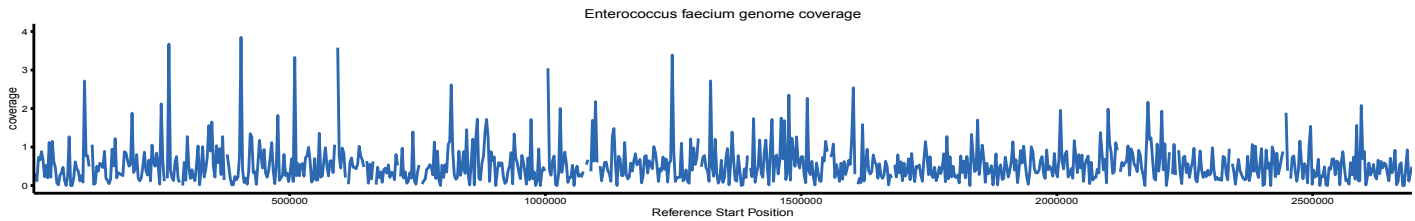

**B**

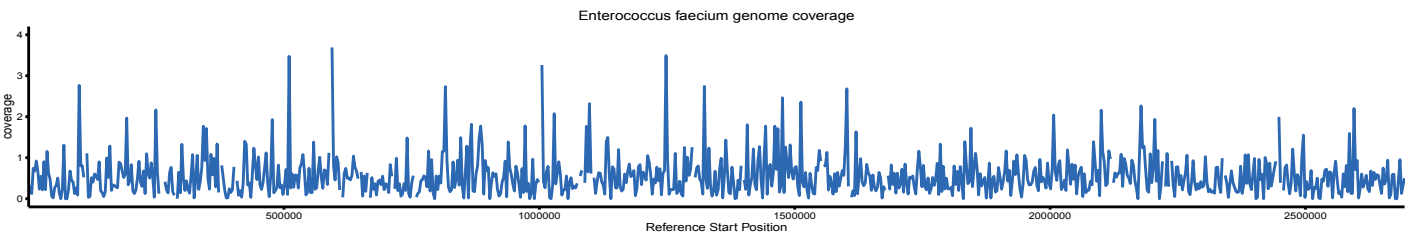

**C**

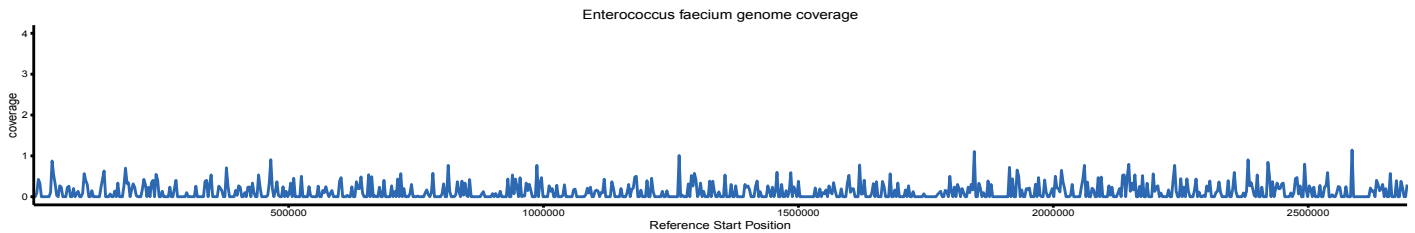

**D**

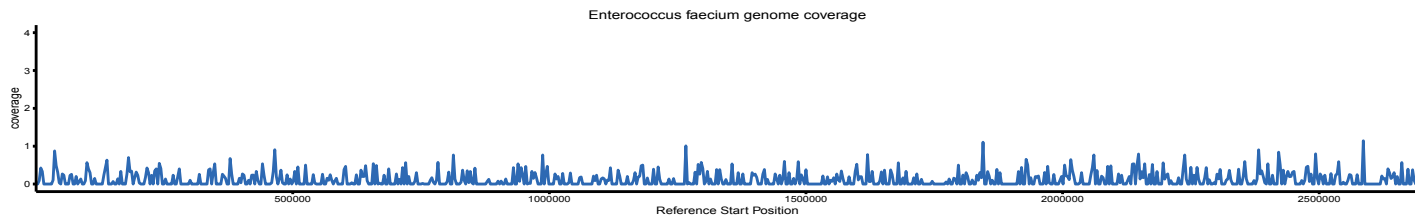

**E**

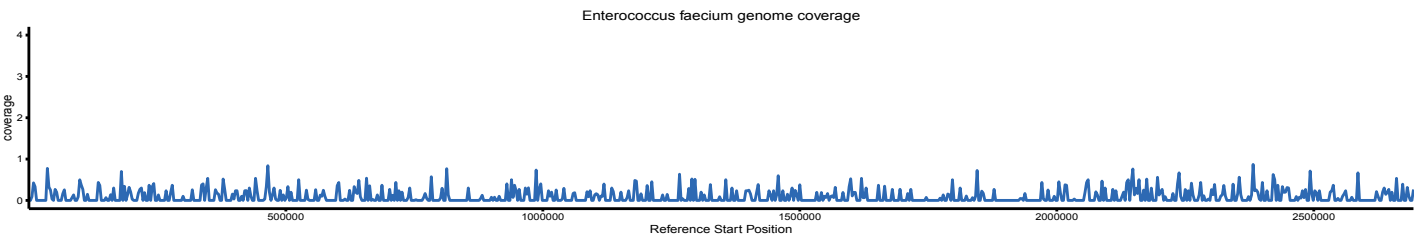

**F**

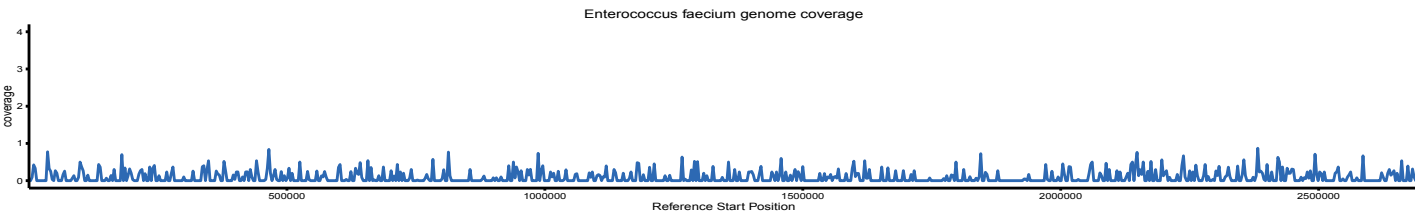

Supplement: Additional file 14: Figure S9. — Mapping of Enterococcus faecium identified reads on the genome of Enterococcus faecium DO (NC_017960.1) using different minimum identity values while mapping. a Minimum identity of 65 %; b minimum identity of 70 %; c minimum identity of 85 %; d minimum identity of 90 %; e minimum identity of 95 %; f minimum identity of 100 %. (PDF 382 kb) [file 13073_2016_326_MOESM14_ESM.pdf]

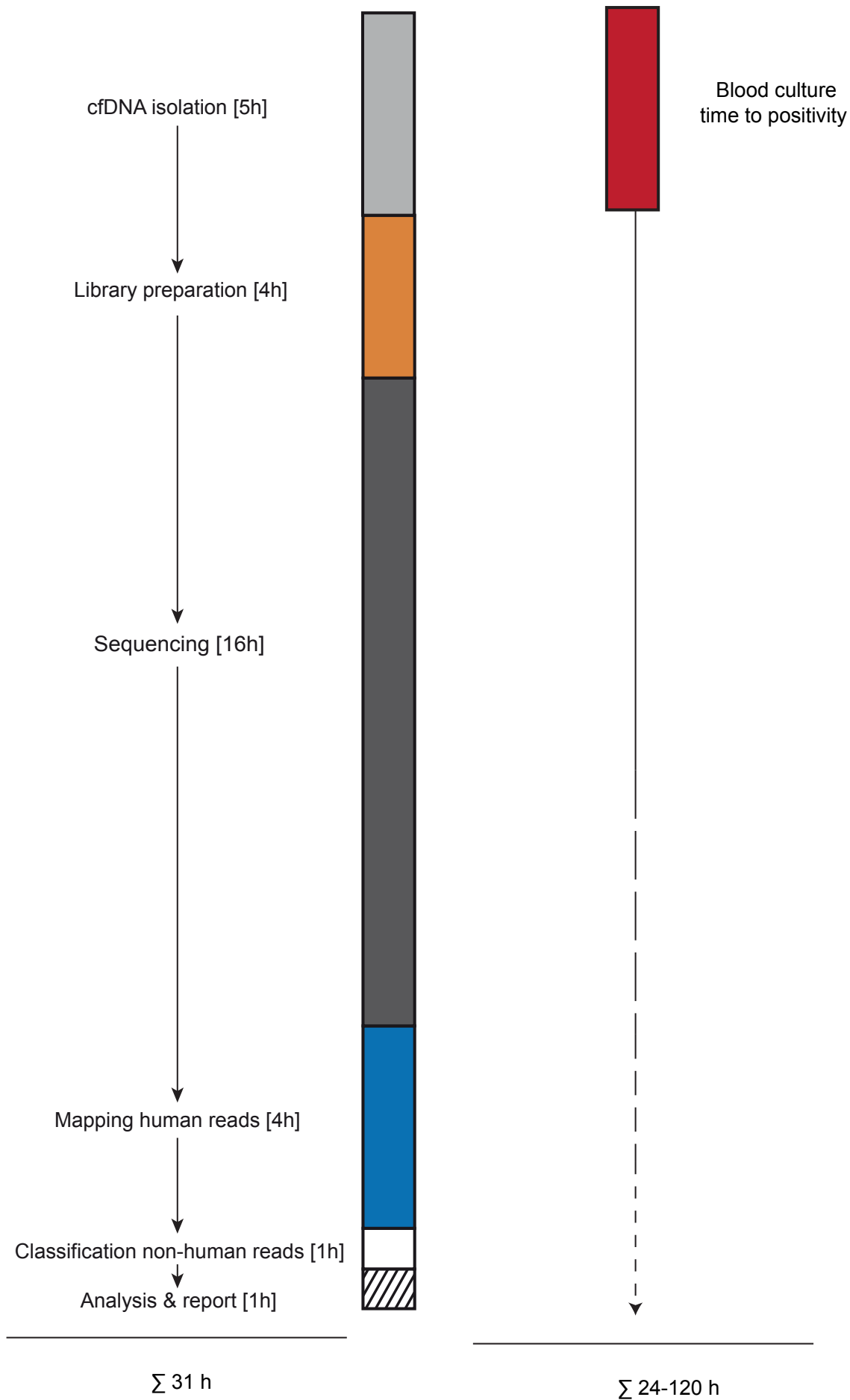

Supplement: Additional file 15: Figure S8. — Workflow and time distribution of NGS-based pathogen identification and blood culture. Flow chart and bar chart of the individual steps and the time required for the NGS-based identification of bacteremia-causing species and conventional blood culture. As time to positivity varies substantially, a time frame of 24 to 120 h is given for blood culture. (PDF 42 kb) [file 13073_2016_326_MOESM15_ESM.pdf]
