# Supplementary material for: Next-generation sequencing diagnostics of bacteremia in septic patients
Source: Genome Med. 2016 Jul 1;8:73. doi: 10.1186/s13073-016-0326-8 (PMC4930583; doi:10.1186/s13073-016-0326-8)
Supplement: Additional file 5: Figure S3. — Distribution of species-specific normalized read counts in septic patients and controls for potential contaminant species. Red, septic patients; blue, controls (elective surgery (timepoint T0) and healthy volunteers). (PDF 18 kb) [file 13073_2016_326_MOESM5_ESM.pdf]

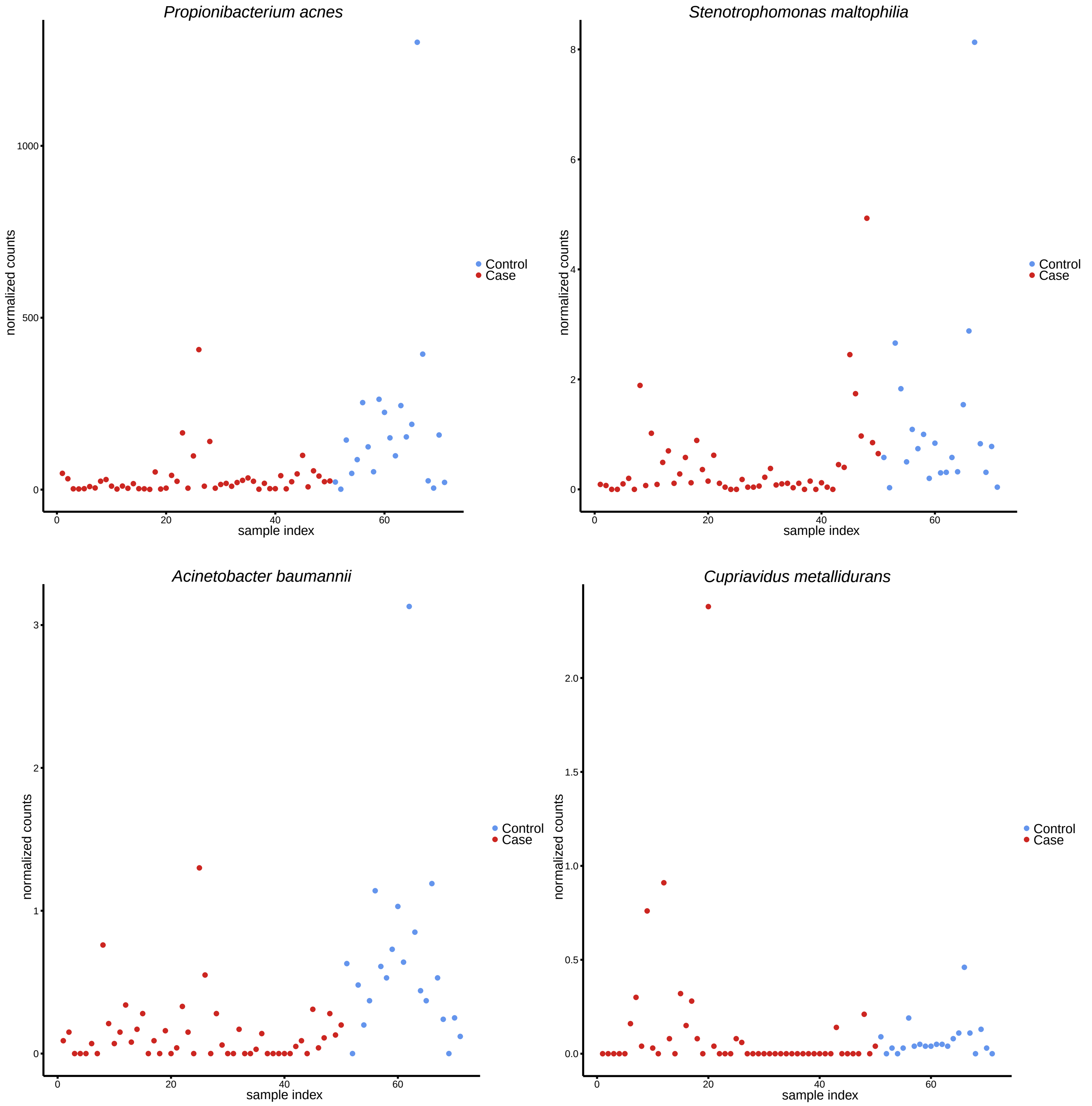
Propionibacterium acnes
normalized counts
sample index
Control
Case
Stenotrophomonas maltophilia
normalized counts
sample index
Control
Case
Acinetobacter baumannii
normalized counts
sample index
Control
Case
Cupriavidus metallidurans
normalized counts
sample index
Control
Case
